# Disruption of Male *Spodoptera litura* Pheromone Attraction by Structurally Related Compounds: Electrophysiological and Field Evidence

**DOI:** 10.3390/insects17101069

**Published:** 2026-10-16

**Authors:** Seon Ah Jeong, Jong Hwan Shin, Soon Hwa Kwon, Bong-Kyu Byun, Kye Chung Park

**Affiliations:** 1 Citrus Research Center, Department of Horticultural Crop Research, National Institute of Horticultural and Herbal Science, Jeju 63607, Republic of Korea; jeong420@korea.kr (S.A.J.); sjh5576@korea.kr (J.H.S.); shkwonn@korea.kr (S.H.K.); 2 Department of Biological Science & Biotechnology, Hannam University, Daejeon 34430, Republic of Korea; bkbyun@hnu.kr; 3 New Zealand Institute for Bioeconomy Science, Christchurch 8140, New Zealand

**Keywords:** field trapping, GC-EAD, kairomones, olfactory receptor neuron, olfactory sensilla, pheromone antagonist, sex pheromone

## Abstract

The tobacco cutworm, *Spodoptera litura*, is a globally important agricultural pest that relies on a female-produced sex pheromone blend for mate location. Here, we investigated the electrophysiological and behavioral responses of *S. litura* to its sex pheromone components and structurally related compounds using gas chromatography–electroantennogrm detection (GC-EAD) and field trapping assays. Most of the 17 compounds tested elicited antennal responses in both sexes, although response magnitude and sensitivity differed markedly among compounds and between males and females. Male responses were generally stronger, but females also detected several pheromone-related compounds with high sensitivity, demonstrating previously unrecognized pheromone autodetection in *S. litura*. In field assays, four compounds, Z7-12:Ac, Z9-14:Ac, Z9-14:OH, and Z9E12-14:Ac, strongly inhibited male attraction to the conspecific pheromone. Other structurally related compounds either had weaker or inconsistent inhibitory effects. Our findings suggest that broad sensitivity to structurally related and heterospecific pheromone compounds may contribute to discrimination among chemically similar odor signals and thereby facilitate species recognition and reproductive isolation in *Spodoptera*. The potent behavioral antagonists identified here also provide promising candidates for the development of pheromone-based, environmentally sustainable approaches to *S. litura* management.

## Simple Summary

The tobacco cutworm, *Spodoptera litura*, is a major agricultural pest that damages a wide range of crops worldwide. Male moths use sex pheromones released by females to locate mates. Understanding how moths respond to pheromone signals may provide new opportunities to disrupt mating and control pest populations without relying heavily on chemical insecticides. In this study, we investigated the responses of male and female *S. litura* to the female sex pheromone and structurally related compounds using electrophysiological analysis and field trapping experiments. We identified several compounds that strongly interfered with male attraction to the female sex pheromone. These findings provide a basis for developing environmentally friendly control strategies that disrupt mating behavior and reduce reliance on conventional chemical insecticides.

## 1. Introduction

Chemical communication is fundamental to insect survival and reproduction, mediating behaviors ranging from mate location and oviposition to host selection and foraging [1]. Insects detect chemical cues primarily through olfactory receptor neurons (ORNs) housed in specialized sensilla, particularly on the antennae [2]. The organization and response properties of these sensory neurons are often highly specialized, allowing insects to distinguish among chemically related odorants and to extract biologically relevant information from complex odor environments. Such olfactory specialization can have important consequences for host selection and reproductive isolation, particularly when closely related species encounter overlapping chemical signals [3,4,5,6].

Sex pheromones provide a particularly well-characterized example of this sensory specialization. In moths, females release species-specific blends of volatile compounds that are detected by males and guide long-range mate location. Pheromone communication depends not only on the chemical identity of individual components but also on their relative proportions, concentration, and spatial and temporal distribution within an odor plume. Male moths possess specialized ORNs that respond strongly to conspecific pheromone components, but they can also detect structurally related compounds that are not components of the conspecific blend. Such compounds may include pheromone components of closely related or sympatric species and can alter pheromone-guided behavior by enhancing, reducing, or terminating upwind attraction [5,7,8,9,10].

This capacity to detect chemically related compounds may be particularly important for species recognition. In several moth taxa, pheromone-guided behavior is influenced by the combinatorial activity of sensory pathways responding to attractive pheromone components and pathways responding to compounds that inhibit or modify attraction [11]. Compounds that enhance attraction at one concentration or blend ratio can also act as antagonists at another concentration or in a heterospecific context [12,13]. Thus, pheromone communication is not necessarily encoded by a simple response to a single attractant; rather, behavioral decisions can emerge from the integration of multiple olfactory signals. Detection of heterospecific pheromone components may consequently provide males with information that helps distinguish conspecific from heterospecific odor sources and thereby contributes to reproductive isolation [5].

The tobacco cutworm, *Spodoptera litura* (Lepidoptera: Noctuidae), provides an excellent system to investigate these processes. *S. litura* is a highly polyphagous and widely distributed agricultural pest that feeds on more than 300 plant species from over 40 families [14]. Its major hosts include tobacco, soybean, cotton, cabbage, spinach, peanut, tomato, and eggplant, as well as numerous other vegetable and ornamental crops, including citrus [15,16,17]. The broad host range and widespread distribution of *S. litura* increase the likelihood of ecological and chemical overlap with other pest species, including closely related *Spodoptera* species that share host plants and geographic ranges [18,19].

The female sex pheromone of *S. litura* was first characterized from a Japanese population as a blend containing Z9E11-14:Ac, Z9E12-14:Ac, Z9-14:Ac, and E11-14:Ac, with behavioral studies subsequently demonstrating that Z9E11-14:Ac and Z9E12-14:Ac constitute the principal components responsible for male attraction [20,21,22]. The optimal ratio of these two components has varied among studies and geographic populations. A ratio of approximately 10:1 was reported in early studies [20,21,22], whereas field trapping experiments in Korea indicated an optimal ratio of approximately 19:1 [23]. These differences highlight the importance of pheromone composition and blend ratio in determining behavioral responses in *S. litura*.

Importantly, the pheromone chemistry of *S. litura* overlaps extensively with that of other *Spodoptera* species. Closely related species such as *S. exigua*, *S. frugiperda*, *S. littoralis*, *S. cosmioides*, and *S. eridania* use several structurally similar compounds as sex pheromone components, although their blend compositions and relative proportions differ [24,25,26,27]. Such overlap creates a potential sensory challenge: males must detect a conspecific pheromone signal while avoiding inappropriate responses to chemically similar signals produced by heterospecific females. The mechanisms by which *S. litura* distinguishes these chemically related odorants, and the extent to which structurally related compounds modify pheromone-guided behavior, remain poorly understood.

Electrophysiological approaches provide an effective means of characterizing this olfactory sensitivity [28,29]. Gas chromatography–electroantennogram detection (GC-EAD) can identify compounds that activate antennal ORNs, whereas behavioral assays are required to determine whether antennal detection translates into biologically meaningful responses. Importantly, these two levels of response need not correspond directly: compounds that strongly activate antennae may have little behavioral effect, whereas compounds producing relatively weak antennal responses may nevertheless strongly alter pheromone-guided behavior. Integrating electrophysiological and behavioral measurements can therefore reveal both the breadth of peripheral olfactory sensitivity and the subset of chemical signals that are behaviorally relevant.

Although male moths have traditionally received most attention in studies of sex pheromone perception, females may also detect their own pheromone components and related compounds. Pheromone autodetection has been reported in several moth species [30,31,32,33] but its sensory basis and behavioral significance remain poorly understood. In particular, little is known about the extent to which female *S. litura* can detect conspecific pheromone components or structurally related compounds and whether such sensitivity differs from that of males.

Here, we investigated the electrophysiological and behavioral responses of *S. litura* to its sex pheromone components and a set of structurally related compounds using GC-EAD and field trapping assays. We first characterized antennal responses of male and female moths to 17 pheromone-related compounds to determine the breadth and sex specificity of peripheral olfactory sensitivity. We then evaluated the behavioral effects of these compounds on male attraction to the conspecific pheromone under field conditions, with particular emphasis on compounds that enhanced or inhibited pheromone-mediated attraction. By comparing electrophysiological sensitivity with behavioral activity, we sought to identify chemical features associated with pheromone antagonism and to determine whether compounds related to the pheromones of other *Spodoptera* species can modify mate-attraction behavior in *S. litura*. Together, these experiments provide insight into the chemical and sensory mechanisms underlying pheromone discrimination in *S. litura* and identify behaviorally active compounds with potential for development as semiochemical tools for pest management.

## 2. Materials and Methods

### 2.1. Test Insects

Pupae of *S. litura* were provided by the Rural Development Administration, Republic of Korea. The pupae were distinguished the sex by examining the ventral segment near the end of the abdomen and individually transferred to Petri dishes and placed in a rearing room under 25 °C temperature, 70% relative humidity, and a 14L:10D light conditions. Adult moths were used for GC-EAD experiments within 3 to 4 days after emergence.

### 2.2. Test Materials

Sex pheromone of *S. litura* and structurally related compounds were used in this study. A total of 17 compounds were selected and used in this study (Table 1). These include female sex pheromones of *S. litura* [20,26,34], sex pheromone components of other *Spodoptera* species [26,27,35,36,37], and those with similar chemical structures. These 17 compounds were purchased from Pherobank (Wijk bij Duurstede, The Netherlands), diluted in hexane, and used in both electrophysiology and behavioral studies. Each compound was analyzed by GC to examine its retention time. Based on the GC analysis, two different mixture solutions were prepared in hexane to minimize overlapping retention times as much as possible. Pheromone Mixture A contained nine compounds, whereas Pheromone Mixture B contained eight remaining compounds (Table 1).

### 2.3. Gas Chromatography–Electroantennogram Detection (GC-EAD) Study

Olfactory activities of each test compound on the male and female antennae of *S. litura* were examined by using GC-EAD analysis. A GC (7890A, Agilent, Santa Clara, CA, USA) equipped with a flame ionization detector (FID) and a DB-5 column was used for GC-EAD study. The GC oven was kept at an initial temperature of 60 °C for 1 min and then increased by 10 °C per minute to reach 240 °C and then held at this temperature for 10 min. The split/splitless injector was kept at 250 °C, and the FID was kept at 250 °C. The end of the GC column was connected to an internal two-way splitter (G3180B, Agilent, Santa Clara, CA, USA) and nitrogen gas was used as make-up gas to split the eluent 1: 1, with half of the eluent sent to the FID and the other half to the EAD. To measure the GC-EAD responses, a moth was anesthetized with carbon dioxide gas, the base of the antenna was cut off from the head, the tip of the antenna was slightly severed with a knife, and the base of the antenna was attached to a glass capillary containing electroconductive gel (Spectro 360, Parker Laboratories Inc., Fairfield, NJ, USA) to serve as the reference electrode. The severed tip of the antenna was attached to a glass capillary prepared in the same way and used as the recording electrode. One μl aliquot of a test solution (Mix A or Mix B containing sex pheromone-related compounds) was injected into the GC, and GC-EAD responses were recorded, stored and analyzed by using software (Autospike 32, Syntech, Buchenbach, Germany). The EAD responses were classified into four categories based on their relative strength (largest ones: ++++; close to half: +++; smaller than half: ++; and recognisable but very small: +).

### 2.4. Field Trapping Test

Field trapping tests were conducted to evaluate the behavioral effects of 17 sex pheromone and structurally related compounds on the attraction of male *S. litura* to the conspecific female sex pheromone. Nine agricultural fields located around Jeong-Eup, Jeonbuk Province, Republic of Korea, were selected as experimental sites. The geographic coordinates and major crops present at each site are shown in Table 2.

Red delta traps equipped with replaceable sticky bases were used for all field experiments. Commercial sex pheromone lures for *S. litura*, containing a binary blend of Z9E11-14:Ac and Z9Z12-14:Ac, were obtained from Green Agrotech (Gyeongsan, Republic of Korea). Test compounds were formulated in either polyethylene (PE) bag or rubber septum dispensers. PE bag dispensers were prepared using square PE bags (3 × 3 cm; 100 μm thickness). A piece of pelt impregnated with the appropriate amount of test compound dissolved in hexane was placed inside each PE bag, and the open end was sealed using a pouch sealer (SK-210; Sambo Tech Corporation, Seoul, Republic of Korea). Rubber septum dispensers were prepared by applying an aliquot of a hexane solution containing the appropriate amount of test compound to the septum and allowing the solvent to evaporate under a gentle stream of air, thereby allowing the compound to penetrate the rubber matrix. Prepared dispensers were individually stored in airtight bags (aluminium foil laminate heat sealer bag with PE inner lining) at −20 °C until use.

For field trapping tests, each test compound was evaluated at a dose of 10 μg per dispenser. This amount was chosen based on a preliminary test. A dispenser containing the test compound was placed at the center of a sticky base and positioned inside a delta trap containing a standard *S. litura* sex pheromone lure. The pheromone lure and test-compound dispenser were maintained as separate odor sources within the same trap, with an approximate distance of 7 cm between the two dispensers. Traps were suspended approximately 1.5 m above the ground. The species and number of moths captured in each trap were recorded at intervals of approximately 1–7 days, depending on field conditions and experimental schedule. Pheromone lures were replaced with new ones in two to four-week intervals.

Two experimental series were conducted to evaluate the behavioral effects of the 17 compounds. In Trial I, test compounds were released from PE bag dispensers, whereas in Trial II, the same compounds were released from rubber septum dispensers. In both trials, each test-compound dispenser contained 10 μg of the respective compound and was evaluated in combination with a standard *S. litura* sex pheromone lure. The pheromone lure alone served as the control treatment. Traps containing different treatments were deployed in the experimental fields, and trap catches were recorded throughout the experimental period. The location of traps was randomized, and Trials I and II were replicated eight and nine times, respectively, across nine test fields.

Seasonal occurrence of adult *S. litura* was monitored at the nine study sites from May to November 2023. At each site, a delta trap containing a rubber septum dispenser loaded with the *S. litura* female sex pheromone blend was installed and maintained throughout the monitoring period. Traps were inspected and serviced at regular intervals, and captured moths were identified to species and sex by visual examination. The number of adult *S. litura* captured at each site was recorded to characterize seasonal and site-specific variation in adult abundance.

Trap catches from the field bioassays were used to compare the attraction of male *S. litura* to the standard sex pheromone in the presence or absence of each test compound. Data were analyzed by analysis of variance (ANOVA), followed by Tukey’s HSD test for pairwise comparisons among treatments. Statistical significance was evaluated at *p* < 0.05.

## 3. Results

### 3.1. Electrophysiological Responses to Sex Pheromone-Related Compounds

**Figure 1:**
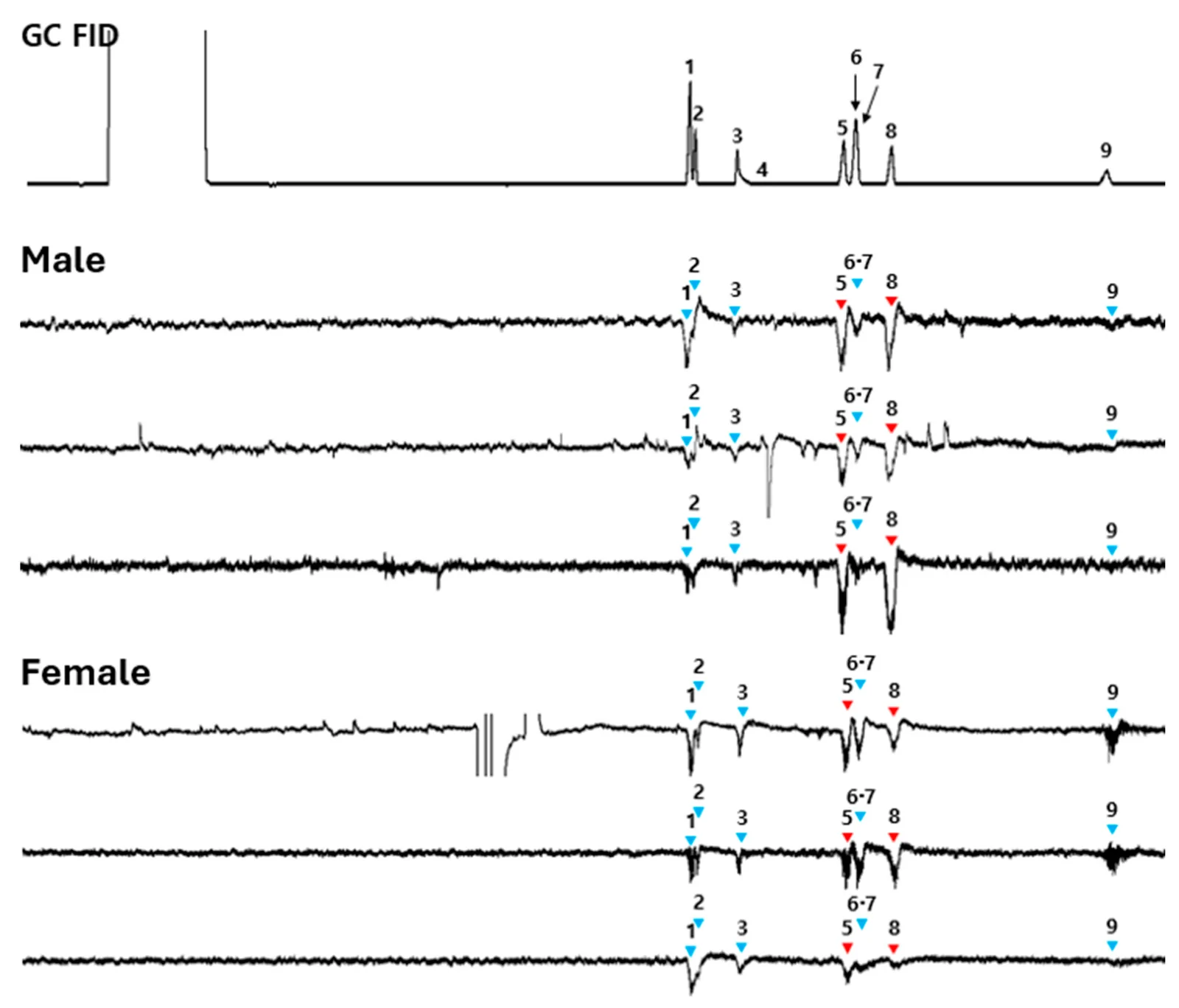
GC-EAD (gas chromatography–electroantennogram detection) responses of male and female *Spodoptera litura* to test mixture A containing nine sex pheromone-related compounds (compounds 1–9). Arrowheads and corresponding numbers in the EAD traces indicate visible responses; red arrowheads denote responses to conspecific sex pheromone compounds, whereas blue arrowheads indicate responses to non-pheromone compounds (1: *Z*7-12:Ac; 2: *Z*9-14:Ald; 3: *Z*9-14:OH, 4: *E*7-12:Ac; 5: *Z*9-14:Ac; 6: *Z*11-16:Ald; 7: *Z*11-14:Ac; 8: *Z*9*E*11-14:Ac; 9: *Z*11-16:Ac).

**Figure 2:**
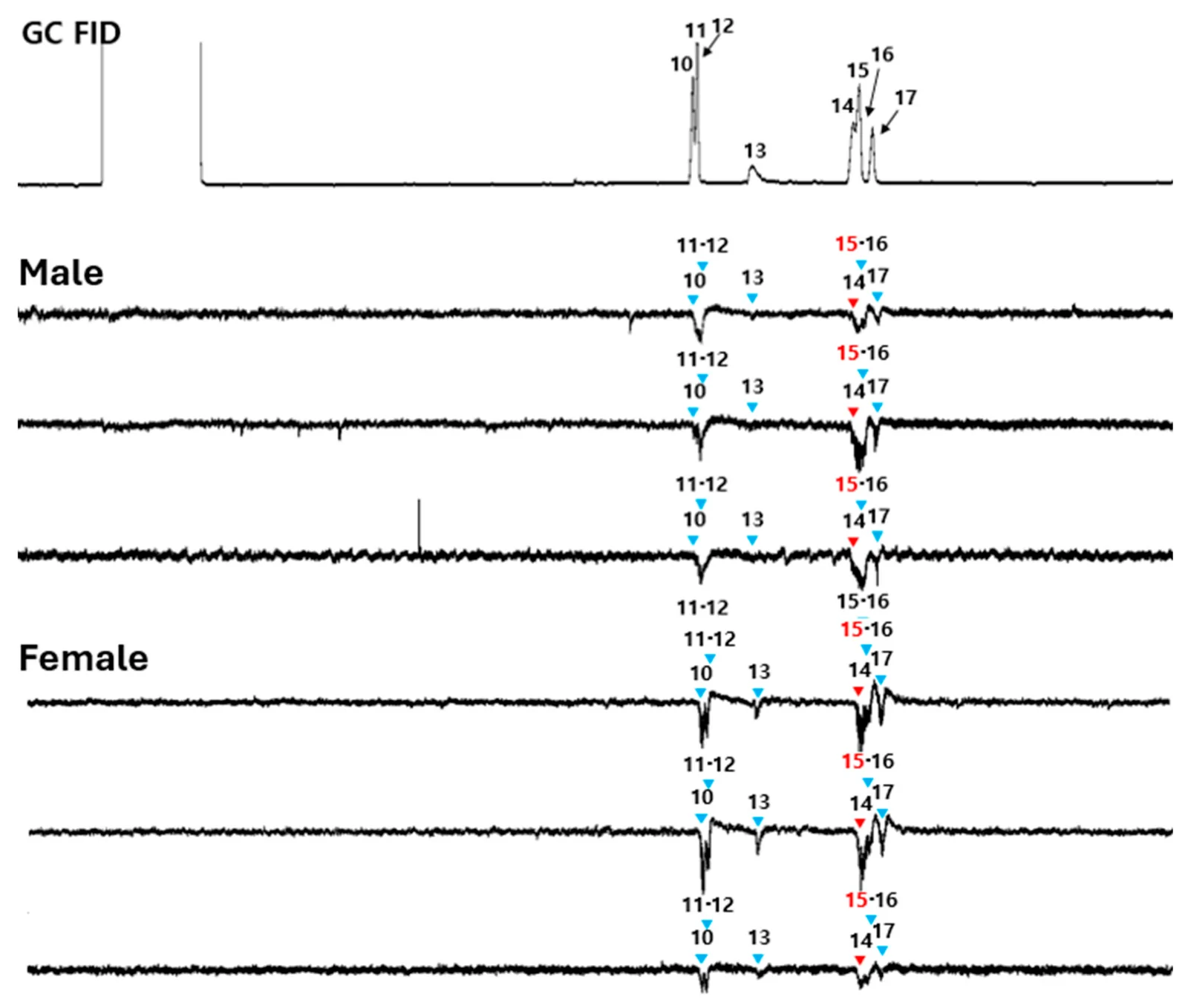
GC-EAD (gas chromatography–electroantennogram detection) responses of male and female *Spodoptera litura* to test mixture B containing eight sex pheromone-related compounds (compounds 10–17). Arrowheads and corresponding numbers in the EAD traces indicate significant responses; red arrowheads denote responses to conspecific sex pheromone compounds, whereas blue arrowheads indicate responses to non-pheromone compounds (10: 11-12:Ac; 11: 12:Ac; 12: *Z*9-12:Ac; 13: *Z*9*E*12-14:OH; 14: *E*11-14:Ac; 15: *Z*9*E*12-14:Ac; 16: *Z*9*Z*12-14:Ac; 17: 14:Ac).

GC-EAD analysis was used to characterize the antennal responses of male and female *S. litura* to 17 sex pheromone and structurally related compounds. Sixteen of the 17 compounds elicited reproducible EAD responses from male *S. litura*, whereas E7-12:Ac did not produce a detectable response (Figure 1 and Figure 2; Table 3). The magnitude of male antennal responses varied substantially among compounds.

Female *S. litura* also exhibited EAD responses to most of the compounds that elicited responses in males, although response magnitudes differed among compounds and between sexes (Figure 1 and Figure 2; Table 3). Differences between male and female EAD responses were observed for 10 compounds: Z9-14:Ald, Z9-14:OH, Z11-16:Ald, Z11-14:Ac, Z9E11-14:Ac, 11-12:Ac, 12:Ac, E11-14:Ac, Z9E12-14:Ac, and Z9Z12-14:Ac (Table 3). The largest sex-specific differences were observed for Z9E11-14:Ac, 11-12:Ac, and E11-14:Ac. Z9E11-14:Ac elicited a substantially stronger response in males, whereas 11-12:Ac and E11-14:Ac elicited stronger responses in females. The two alcohols, Z9-14:OH and Z9E12-14:OH, elicited relatively weak responses in both sexes.

### 3.2. Seasonal Occurrence of Spodoptera litura in Field Sites

**Figure 3:**
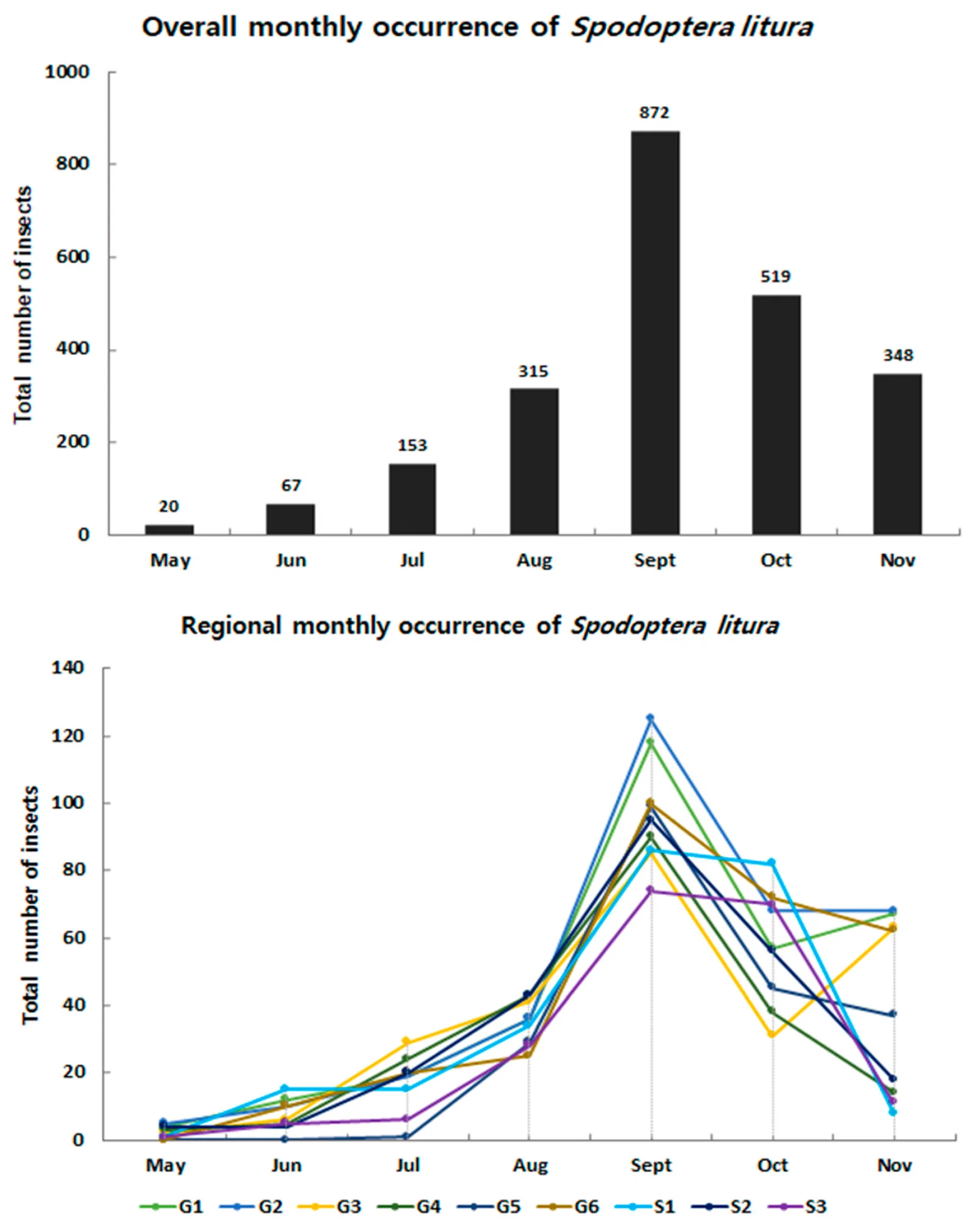
Monthly occurrence of *Spodoptera litura* at nine cropping fields in Jeongeup, Republic of Korea, in 2023, based on the number of male moths captured in sex pheromone-baited delta traps. Top: total monthly captures; bottom: monthly trap catches by location (see Table 2 for test field codes).

Adult male *S. litura* populations were monitored continuously from May to November 2023 using sex pheromone-baited delta traps at nine agricultural fields in the Jeong-Eup region of Jeonbuk Province, Republic of Korea. A total of 2294 male *S. litura* were captured during the monitoring period (Figure 3). Only male *S. litura* were captured in the pheromone-baited traps, and no female *S. litura* were captured in the traps baited with pheromone-only or pheromone plus test compounds.

Monthly trap catches varied substantially throughout the season, with the highest total catch recorded in September, which accounted for 38% of the total number of moths captured during the monitoring period (Figure 3). Population trends were broadly similar among the nine fields until early September. Thereafter, substantial differences in trap catches among fields became apparent. By November, fewer than 20 moths were captured at each of four sites (G4, S1, S2, and S3), whereas more than 60 moths were captured at each of three other sites (G1, G2, and G3) (Figure 3).

### 3.3. Behavioral Effects of Pheromone-Related Compounds on Male Attraction

**Figure 4:**
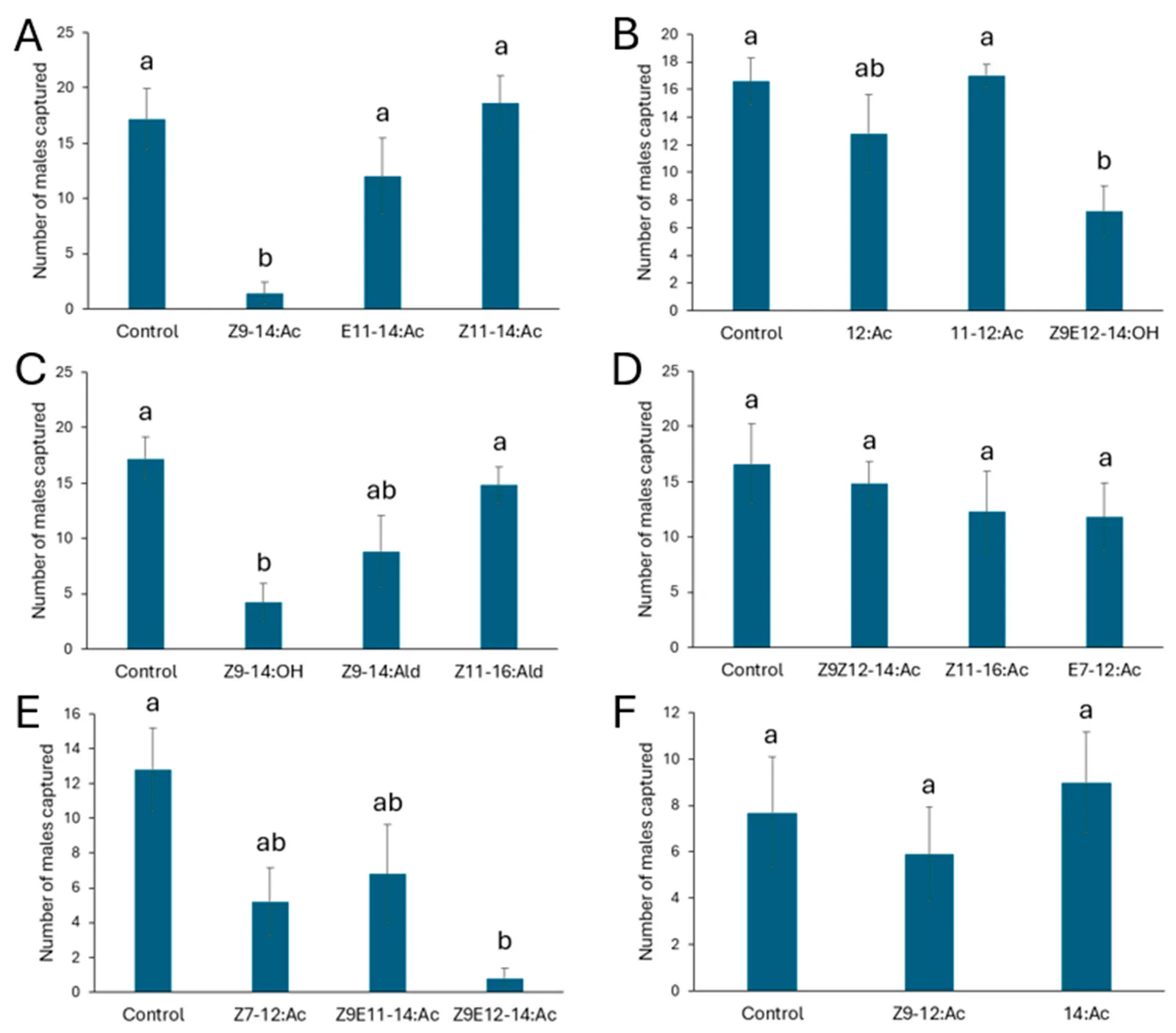
Effects of pheromone-related compounds on the attraction of male *Spodoptera litura* to conspecific female sex pheromone, measured as the actual number of males captured in delta traps. Traps were baited with sex pheromone alone (control) or sex pheromone plus a polyethylene (PE) dispenser containing 10 μg of each test compound across six experimental sets (**A**–**F**). Field trials were conducted in Jeongeup, Republic of Korea, in 2023. Different letters indicate significant differences (ANOVA followed by Tukey’s HSD, *p* = 0.05; mean ± SE, *n* = 8).

**Figure 5:**
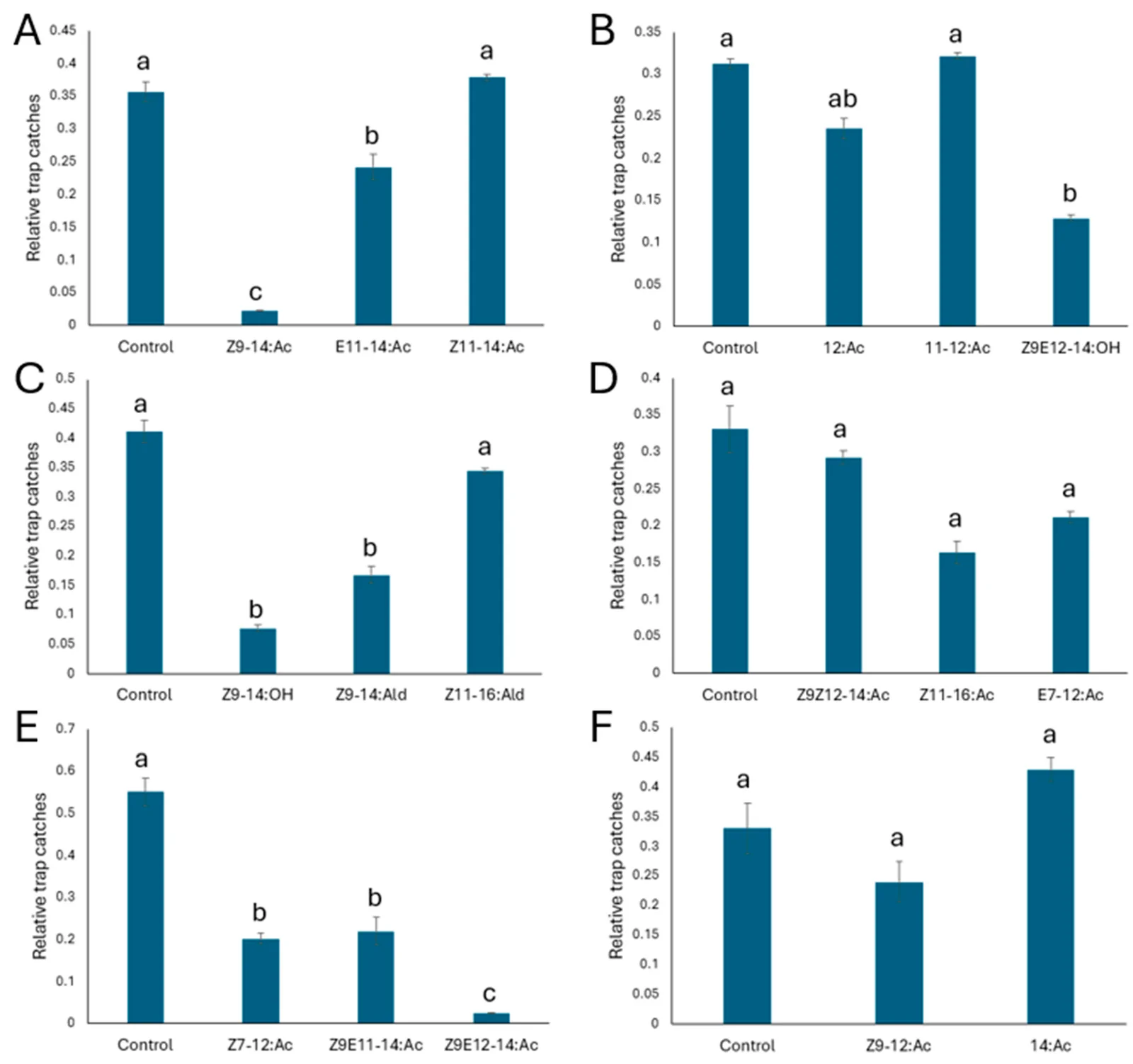
Effects of pheromone-related compounds on the attraction of male *Spodoptera litura* to conspecific female sex pheromone, expressed as normalized trap catches in delta traps. Treatments included sex pheromone alone (control) and sex pheromone plus a polyethylene (PE) dispenser containing 10 μg of each test compound across six experimental sets (**A**–**F**). Field trials were conducted in Jeongeup, Republic of Korea, in 2023. Different letters indicate significant differences (ANOVA followed by Tukey’s HSD, *p* = 0.05; mean ± SE, *n* = 8).

**Figure 6:**
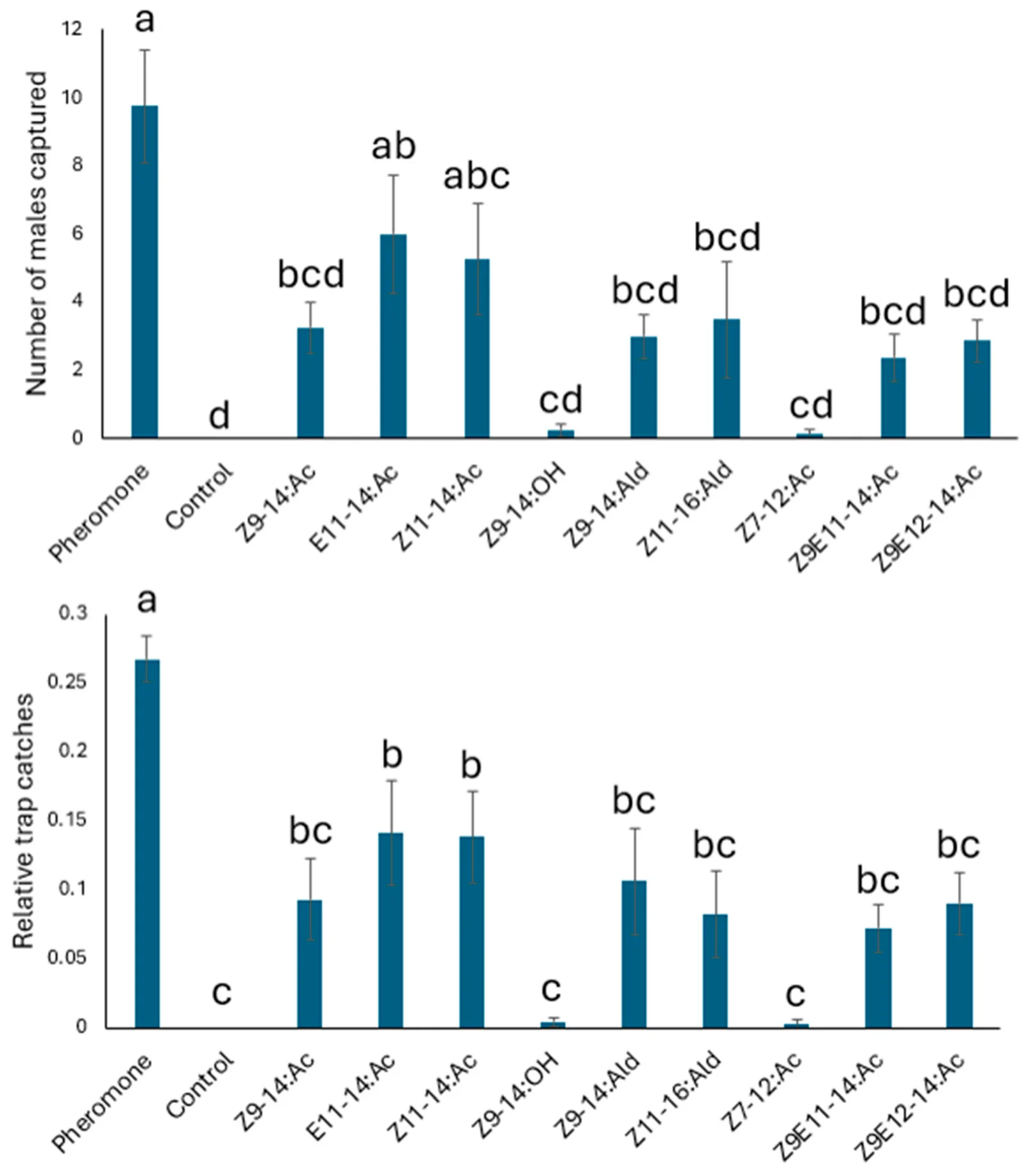
Effects of pheromone-related compounds on the attraction of male *Spodoptera litura* to conspecific female sex pheromone, shown as the actual number (top) and normalized proportion (bottom) of males captured in delta traps. Treatments included a positive control (sex pheromone), a negative control (blank rubber septum), and nine treatments comprising a sex pheromone lure plus a rubber septum dispenser containing 10 μg of each test compound. Field trapping was conducted in Jeongeup, Republic of Korea, in 2023. Different letters indicate significant differences among treatments (ANOVA followed by Tukey’s HSD, *p* = 0.05; mean ± SE, *n* = 9).

The behavioral effects of the 17 pheromone and structurally related compounds were evaluated in field trapping experiments using PE bag and rubber septum dispensers. Several compounds reduced male attraction to traps containing the standard *S. litura* sex pheromone lure, whereas no consistent enhancement of trap catches was detected (Figure 4, Figure 5 and Figure 6). Similar patterns were observed when trap catches were presented as absolute numbers of captured males or as values normalized relative to the corresponding control treatment (Figure 4, Figure 5 and Figure 6).

When the compounds were released from PE bag dispensers, nine compounds, Z9-14:Ac, E11-14:Ac, Z9-14:OH, Z9-14:Ald, Z11-16:Ald, Z7-12:Ac, Z9E11-14:Ac, Z9E12-14:Ac, and Z9E12-14:OH, significantly reduced attraction to the standard pheromone lure (Figure 4 and Figure 5). Among these compounds, Z9-14:Ac and Z9E12-14:Ac produced the strongest inhibitory effects, each reducing trap catches by more than 90% relative to the pheromone-only control (Figure 4A,E and Figure 5A,E). E11-14:Ac, Z9E12-14:OH, Z9-14:OH, Z9-14:Ald, Z7-12:Ac, and Z9E11-14:Ac produced weaker inhibitory effects when released from PE bag dispensers (Figure 4 and Figure 5).

When the test compounds were released from rubber septum dispensers, Z9-14:OH and Z7-12:Ac produced the strongest inhibition, each reducing trap catches by more than 90% relative to the pheromone-only control (Figure 6). Seven additional compounds, Z9-14:Ac, E11-14:Ac, Z11-14:Ac, Z9-14:Ald, Z11-16:Ald, Z9E11-14:Ac, and Z9E12-14:Ac, also significantly reduced male attraction, although their inhibitory effects were less pronounced than those of Z9-14:OH and Z7-12:Ac (Figure 6).

The magnitude of inhibition therefore differed among compounds and between dispenser types. Z9-14:Ac and Z9E12-14:Ac were particularly effective when released from PE bag dispensers, whereas Z9-14:OH and Z7-12:Ac produced the strongest inhibition when released from rubber septum dispensers.

In contrast to males, no female *S. litura* were captured in traps baited with the pheromone alone or the pheromone in combination with the test compounds.

## 4. Discussion

### 4.1. Pheromone Antagonists in Spodoptera litura

Our results demonstrate that several aliphatic compounds structurally related to the female sex pheromone components Z9E11-14:Ac and Z9Z12-14:Ac can interfere with pheromone-mediated attraction of male *S. litura*. Nine compounds exhibited behavioral inhibition, but Z9-14:Ac, Z9-14:OH, and Z9E12-14:Ac showed significant inhibitory activity consistently across all field-trapping experiments in which they were tested. Moreover, Z7-12:Ac, Z9-14:Ac, Z9-14:OH, and Z9E12-14:Ac each reduced trap catches by more than 90% in at least two independent field experiments. These compounds therefore represent particularly promising candidates for investigating the mechanisms and potential applications of pheromone antagonism in *S. litura*.

The behavioral activity of these compounds provides insight into the structural features that determine olfactory-mediated responses in *S. litura*. The strong inhibition produced by the monoenes Z7-12:Ac, Z9-14:Ac, and Z9-14:OH suggests that the position of the double bond and the carbon-chain length contribute to antagonist activity. The terminal functional group also appears to be important. Z9-14:Ac and Z9-14:OH strongly inhibited pheromone attraction, whereas the corresponding aldehyde, Z9-14:Ald, did not. Likewise, Z9E12-14:Ac strongly inhibited attraction, whereas Z9E12-14:OH did not produce a comparable response. Thus, behavioral activity cannot be explained solely by hydrocarbon chain length or double-bond position; the chemical identity of the terminal functional group also appears to determine whether a structurally related compound functions as a behavioral antagonist.

Strong inhibition of pheromone-mediated attraction by structurally related compounds has been reported in several other moth species, including *Cydia pomonella*, *Coleophora laricella*, *Epiphyas postvittana*, and *Plutella xylostella* [11,38,39,40,41]. In some cases, compounds that inhibit attraction in one species are themselves components of the pheromone blends of closely related species [42]. Such observations support the idea that moth pheromone communication systems are shaped not only by sensitivity to conspecific pheromone components but also by the ability to detect chemically similar compounds that provide information about alternative or heterospecific odor sources. The present results extend this concept to *S. litura* by identifying several structurally related compounds that strongly suppress pheromone-mediated attraction under field conditions.

Previous studies provide further evidence that the spatial relationship between pheromone and antagonist sources can strongly influence behavioral responses. In *C. pomonella*, for example, inhibition by a behavioral antagonist occurred when the antagonist and conspecific pheromone were emitted from the same point source, whereas increasing the crosswind separation between the two sources restored attraction to the conspecific pheromone [38]. Similarly, inhibition of male *E. postvittana* by a pheromone-component isomer decreased rapidly as the distance between the inhibitor and pheromone source increased [40]. Together, these observations suggest that moths can use fine-scale spatial and temporal information within odor plumes when evaluating chemically complex pheromone signals.

Our findings therefore indicate that pheromone antagonism in *S. litura* depends on both the molecular structure of the interfering compound and its spatial relationship to the pheromone source. The strong and reproducible inhibition produced by Z7-12:Ac, Z9-14:Ac, Z9-14:OH, and Z9E12-14:Ac makes these compounds particularly suitable for further studies aimed at determining their sensory and neural mechanisms of action and evaluating their potential for pheromone-based management.

### 4.2. Chemical Structures of Pheromone-Related Compounds and Antennal Responses

As expected, and consistent with previous studies [43] by Kawasaki, the two known female sex pheromone components, Z9E11-14:Ac and Z9Z12-14:Ac, elicited strong antennal responses in male *S. litura*. Several structurally related compounds were also detected by the male antennae, although response magnitude varied markedly among compounds. This variation indicates that the peripheral olfactory system can discriminate among compounds that differ only in relatively subtle structural features.

The terminal functional group appeared to be an important determinant of antennal sensitivity. In general, the alcohols and aldehydes with 12- and 14-carbon chains elicited weaker responses than their corresponding acetates, suggesting that functional-group identity influences molecular recognition by antennal olfactory receptors. Geometric configuration was also critical. For example, Z7-12:Ac elicited a strong antennal response, whereas its E-isomer, E7-12:Ac, produced no detectable response. Thus, male *S. litura* antennae can distinguish closely related compounds according to both functional-group identity and double-bond geometry.

These findings are consistent with the highly selective nature of moth pheromone olfactory systems, in which relatively small structural changes can substantially alter receptor activation. At the same time, the broad range of compounds detected in the present study suggests that pheromone-sensitive olfactory systems in *S. litura* are not restricted exclusively to the compounds used in the conspecific pheromone blend. Such broad peripheral sensitivity may provide a sensory basis for detecting chemically related odorants encountered in the environment, including pheromone components of sympatric or closely related species.

### 4.3. Comparison Between Antennal Sensitivity and Behavioral Responses

A general correspondence was observed between antennal sensitivity and behavioral activity: compounds that produced strong EAD responses often exhibited pronounced behavioral effects. For example, Z7-12:Ac, Z9-14:Ac, and Z9E12-14:Ac produced among the strongest antennal responses and also showed strong behavioral inhibition. However, this relationship was not absolute. Most notably, Z9-14:OH produced one of the weakest antennal responses yet strongly inhibited pheromone-mediated attraction. Conversely, several compounds that elicited substantial antennal responses produced little or no detectable behavioral effect. Thus, EAD sensitivity provides information about peripheral detection but does not, by itself, predict the behavioral significance of an odorant.

This distinction was particularly evident for the saturated 12- and 14-carbon acetates, which elicited relatively strong antennal responses but did not significantly affect male behavior. Detection of an odorant at the antenna therefore does not necessarily imply that the compound will be incorporated into the neural representation that controls pheromone-guided behavior. Behavioral output may depend on subsequent processes including receptor-neuron tuning, combinatorial coding, central nervous system processing, concentration-dependent integration, and interactions among excitatory and inhibitory olfactory inputs. The lack of behavioral activity of saturated compounds despite their antennal detectability further suggests that the presence, position, and geometry of double bonds are important determinants of the behavioral interpretation of pheromone-related odors in *S. litura*.

The particularly strong behavioral inhibition produced by Z9-14:OH despite its comparatively weak EAD response is noteworthy. This result cautions against using antennal response magnitude as a direct proxy for behavioral potency and highlights the value of combining electrophysiological and behavioral approaches when identifying pheromone antagonists. Compounds with modest antennal responses may nevertheless exert strong effects if they activate highly behaviorally relevant receptor pathways or inhibitory neural circuits.

### 4.4. Response of Females to Sex Pheromone and Related Compounds

Our results demonstrate that female *S. litura* detect their own sex pheromone components as well as a range of structurally related compounds. Such pheromone autodetection has been reported in some *Spodoptera* [31,32] and other groups of moths [30,33], suggesting that the ability of females to detect conspecific sex pheromone may be more widespread than previously recognized. This possibility is particularly relevant because electrophysiological studies of moth pheromone communication have historically focused predominantly on males, potentially leaving substantial sex-specific olfactory capacity undocumented.

Although the EAD responses were not statistically analyzed, several patterns were apparent. Female *S. litura* generally exhibited weaker EAD responses to the two conspecific pheromone components, Z9E11-14:Ac and Z9Z12-14:Ac, than males, suggesting lower peripheral sensitivity to these compounds. Nevertheless, the response profiles indicated clear sex-related differences in olfactory sensitivity. While males generally exhibited stronger responses to most of the compounds tested, some compounds elicited stronger responses in females; notably, 11-12:Ac and E11-14:Ac produced substantially larger responses in females than in males. The biological significance of these sex-specific differences remains unclear, and behavioral experiments will be required to determine whether these compounds have functional effects on female behavior.

Despite their antennal sensitivity, none of the EAD-active compounds tested elicited a clear behavioral response in females. Thus, females could detect their own pheromone components and several related compounds at the antennal level without exhibiting the behavioral attraction observed in males. This divergence between peripheral detection and behavioral output further emphasizes that antennal sensitivity does not necessarily predict behavioral relevance. Olfactory signals may undergo substantial processing and filtering within the central nervous system before they are translated into behavior.

Interpretation of the female behavioral results is also limited by the absence of an established female behavioral positive control. Whereas the standard sex pheromone provided an appropriate positive control for male attraction assays, no compound or behavioral context was available that reliably elicited attraction in females. Consequently, the absence of behavioral responses should not be interpreted as evidence that the detected compounds are behaviorally irrelevant to females. Future experiments using ecologically relevant behavioral paradigms, such as oviposition-site selection or responses to conspecific female aggregations, may reveal functions that are not captured by the attraction assay used here.

Male *S. litura* possess long trichoid sensilla containing olfactory receptor neurons (ORNs) specialized for the detection of female sex pheromone components and related compounds, whereas females lack this characteristic male-specific sensillum type [44]. The present finding that females nevertheless detect pheromone components raises questions about the peripheral sensory structures and receptor neurons responsible for pheromone autodetection. In males, pheromone detection is well established to involve specialized trichoid sensilla containing ORNs with highly selective responses to individual pheromone components or structurally related compounds [45,46,47,48,49]. In females, however, the cellular basis of pheromone autodetection remains unclear. Single-sensillum recording and receptor-expression studies will therefore be important for identifying the sensillum types and ORN classes responsible for detecting pheromone components and related compounds in female *S. litura*.

Pheromone autodetection could provide females with information about the presence and distribution of conspecifics or heterospecifics. Because *Spodoptera* species may overlap spatially and temporally and may exploit similar host plants, detecting pheromone-related odors could potentially provide information relevant to oviposition-site selection or competition. Female moths have been shown in several species to respond electrophysiologically and behaviorally to their own sex pheromone components, and pheromone autodetection has been proposed to have ecological and reproductive consequences [50,51,52]. Whether pheromone autodetection serves a comparable function in *S. litura*, however, remains to be established experimentally.

### 4.5. Pheromone Communication and Reproductive Isolation in Spodoptera

The broad olfactory sensitivity observed in *S. litura* provides a potential sensory mechanism for distinguishing conspecific pheromone signals from chemically similar signals produced by other *Spodoptera* species. This capacity may be particularly important in environments where multiple congeners occur simultaneously and share host plants or seasonal activity periods. Because successful mate localization depends on accurate recognition of species-specific pheromone signals, sensitivity to heterospecific compounds could help males evaluate the chemical identity of an odor plume before committing to sustained upwind flight.

Studies in other moths and insects have identified ORNs with both overlapping and species-specific response profiles among closely related species [3,53,54]. A comparable organization could occur in *Spodoptera*, with broadly tuned sensory pathways detecting structurally related compounds and more narrowly tuned pathways encoding information that is characteristic of the conspecific pheromone blend. Such an arrangement would allow males to extract information about both the presence of a pheromone source and its chemical identity.

In several moth groups, including heliothine and *Ostrinia* moths, orientation toward conspecific females involves combinatorial input from neurons that promote attraction and neurons that respond to compounds capable of inhibiting or terminating pheromone-mediated flight [5,55,56]. Our behavioral results are consistent with the possibility that *S. litura* similarly integrates excitatory and inhibitory olfactory signals. In particular, compounds that are pheromone components of other *Spodoptera* species but inhibit attraction in *S. litura* could provide sensory information that distinguishes heterospecific from conspecific pheromone plumes. However, the present experiments do not directly demonstrate the existence of antagonist-specific ORNs or central inhibitory circuits. Direct electrophysiological characterization of individual ORNs and neural pathways will be required to test this hypothesis.

This possibility is particularly relevant to *Spodoptera* because closely related species frequently use overlapping sets of structurally similar pheromone compounds but differ in their blend compositions. Reported pheromone blends include Z7-12:Ac, Z9-12:Ac, Z9-14:Ac, and Z11-16:Ac in *S. frugiperda* [25,26,57]; Z9E12-14:Ac, Z9-14:Ac, and Z9-14:OH in *S. exigua* [24,29,35] Z9E11-14:Ac and Z9Z12-14:Ac in *S. litura* [21] Z9E11-14:Ac, Z9-14:Ac, and E11-14:Ac in S. littoralis [58,59]; Z9-14:Ac and Z9E12-14 in *S. cosmioides* [27] and Z9-14:Ac, Z9E12-14:Ac, Z9Z12-14:Ac, Z9E11-14:Ac, and Z11-16:Ac in *S. eridania* [37]. Thus, species-specific information is encoded not simply by the presence or absence of individual compounds, but also by their relative proportions within the pheromone blend.

The GC-EAD results further demonstrate that *S. litura* males detect a broad spectrum of compounds related to their own pheromone components, including compounds that function as pheromone components in other *Spodoptera* species. This broad sensitivity, combined with the strong behavioral inhibition produced by several heterospecific-related compounds, suggests that olfactory detection of chemically similar signals may contribute to species discrimination. Detection of heterospecific pheromone components could reduce inappropriate upwind orientation and mating attempts, thereby increase the efficiency of mate localization and potentially contribute to reproductive isolation.

High sensitivity to heterospecific pheromone components has similarly been documented in other closely related moths, including *Heliothis* and *Helicoverpa* species [60,61,62] and *Agrotis ipsilon* and *A. segetum* [63]. Such cross-species sensitivity has been proposed to contribute to the evolution and maintenance of reproductive isolation [5]. Our results suggest that a similar principle may operate in *Spodoptera*, where overlapping pheromone chemistry creates a strong selective context for discriminating among closely related signals. We propose that reproductive isolation in this group may therefore depend not only on sensitivity to the conspecific pheromone blend but also on the ability to recognize and behaviorally suppress responses to heterospecific pheromone signals.

### 4.6. Practical Applications

The identification of potent pheromone antagonists provides a potential route toward developing new semiochemical tools for the management of *S. litura*, an economically important agricultural and horticultural pest. This is particularly relevant given that *S. litura* damages a wide range of crops beyond the field vegetables examined here, including citrus [64,65,66], indicating that antagonist-based management strategies developed in this study may be applicable not only to vegetable production systems but also to orchard crops such as citrus. Among the compounds evaluated, Z7-12:Ac, Z9-14:Ac, Z9-14:OH, and Z9E12-14:Ac were particularly effective, producing greater than 90% reductions in trap catches in at least two independent field experiments. Their strong activity under field conditions is encouraging because it demonstrates that the antagonistic effects observed in laboratory assays can persist in complex outdoor environments.

The seasonal monitoring conducted in the present study further provides a basis for evaluating the timing of pheromone-based interventions. Adult *S. litura* populations showed multiple seasonal peaks, with major increases occurring during June–July and August–September and, in some fields, a further peak in October. The highest monthly captures occurred in September, consistent with previous surveys conducted in Korean agricultural landscapes [67,68,69]. The occurrence of synchronized adult peaks across most of the nine monitored fields suggests that regional population dynamics may be sufficiently coordinated to permit area-wide monitoring or management strategies.

Nevertheless, substantial local variation was also observed. In particular, the field at S1 (Sinwol-dong 164) maintained relatively high adult abundance after August, unlike the other monitored fields. The presence of multiple crops, including capsicum, rice, blueberry, hemp, and blackberry, may have contributed to this sustained population density by providing a heterogeneous and temporally extended resource base. This observation emphasizes the importance of considering local cropping patterns and habitat composition when developing semiochemical-based management programs.

The potential of pheromone antagonists for direct pest management has been demonstrated in other lepidopteran pests. For example, the antagonist E8E10-12 produced mating-disruption effects comparable to those of the sex pheromone in *C. pomonella* [70], and incorporation of an antagonistic compound into a pheromone blend successfully disrupted mating in *C. nigricana* [71]. Although relatively few studies have evaluated pheromone antagonists as independent management tools, these precedents, together with the strong field activity observed here, indicate that antagonists warrant further investigation as components of *S. litura* management. Several steps are required before practical deployment can be assessed. Future studies should determine the dose–response relationships, release rates, effective source configurations, duration of activity, and optimal spatial deployment of the most potent antagonists. Large-scale mating-disruption experiments will also be necessary to determine whether reductions in trap catches translate into reduced mating, oviposition, and population growth.

Overall, our study identifies a set of structurally related compounds that can strongly interfere with pheromone-mediated attraction in *S. litura* and provides evidence that both molecular structure and odor-source configuration shape this behavioral inhibition. The combination of electrophysiological, behavioral, and field data further suggests that the *S. litura* olfactory system is capable of detecting a chemically diverse set of pheromone-related compounds and using this information to discriminate among odor signals. These findings provide a foundation for understanding the sensory basis of pheromone antagonism and reproductive isolation in Spodoptera and highlight pheromone antagonists as promising candidates for development as semiochemical-based management tools.

## Figures and Tables

**Table 1:** Sex pheromone and structurally related compounds used in this study, including their sources, purity, and grouping into two mixtures (A and B) for GC-EAD experiments.

Compound	Abbreviation	EAD Mix	Source	Purity
Dodecyl acetate	12:Ac	B	Pherobank	>98%
(*E*)-7-Dodecenyl acetate	*E*7-12:Ac	A	Pherobank	>98%
(*Z*)-7-Dodecenyl acetate	*Z*7-12:Ac **	A	Pherobank	>98%
(*Z*)-9-Dodecenyl acetate	*Z*9-12:Ac	B	Pherobank	>98%
11-Dodecenyl acetate	11-12:Ac	B	Pherobank	>98%
(*Z*)-9-Tetradecenal	*Z*9-14:Ald	A	Pherobank	>93%
(*Z*)-9-Tetradecenol	*Z*9-14:OH **	A	Pherobank	>98%
(*Z*,*E*)-9,12-Tetradecadienol	*Z*9*E*12-14:OH **	B	Pherobank	>95%
Tetradecyl acetate	14:Ac	B	Sigma-Aldrich	>98%
(*Z*)-9-Tetradecenyl acetate	*Z*9-14:Ac **	A	Pherobank	>98%
(*E*)-11-Tetradecenyl acetate	*E*11-14:Ac	B	Pherobank	>98%
(*Z*)-11-Tetradecenyl acetate	*Z*11-14:Ac	A	Pherobank	>98%
(*Z*,*E*)-9,11-Tetradecadienyl acetate	***Z*** 9*E*11-14:Ac *	A	Pherobank	>95%
(*Z*,*E*)-9,12-Tetradecadienyl acetate	***Z*9*E*12-14:Ac** *	B	Pherobank	>95%
(*Z,Z*)-9,12-Tetradecadienyl acetate	*Z*9*Z*12-14:Ac **	B	Pherobank	>95%
(*Z*)-11-Hexadecenal	*Z*11-16:Ald	A	Pherobank	>95%
(*Z*)-11-Hexadecenyl acetate	*Z*11-16:Ac **	A	Pherobank	>98%

* Two female sex pheromone components of *Spodoptera litura*. ** Sex pheromone components of other *Spodoptera* species.

**Table 2:** Coordinates of each test field and the main crops cultivated in fields around Jeongeup, Jeonbuk province, Republic of Korea.

Code	Location	Crop Planted	Coordinate
G1	Gyoam-dong 108	peanut	35°32′04.3″ N 126°50′51.1″ E
G2	Gyoam-dong 177	perilla	35°31′28.9″ N 126°50′54.3″ E
G3	Gyoam-dong 207	capsicum, corn, cabbage, raspberry	35°31′22.8″ N 126°50′55.2″ E
G4	Gyoam-dong 314	potato	35°31′28.6″ N 126°50′48.8″ E
G5	Gyoam-dong 460	capsicum, peanut	35°31′57.3″ N 126°50′42.9″ E
G6	Gyoam-dong 515	capsicum, green onion, onion, corn	35°32′02.1″ N 126°50′42.0″ E
S1	Sinwol-dong 164	capsicum, rice, blueberry, hemp, blackberry	35°31′33.4″ N 126°52′00.5″ E
S2	Sinwol-dong 235	capsicum, corn	35°31′43″ N 126°51′56″ E
S3	Sinwol-dong 263	capsicum, green onion, cabbage, the roots of *Aralia continentalis*	35°31′55.3″ N 126°52′03.9″ E

**Table 3:** EAD-activity of sex pheromone-related compounds in test mixture A and B in male and female *Spodoptera litura*. The number of “+” symbols indicates the relative strength of EAD responses to each compound.

Mixture Group	Peak Number	Compound	EAD Responses ^1^	
			Male	Female
A	1	*Z*7-12:Ac	+++	+++
	2	*Z*9-14:Ald	++	+
	3	*Z*9-14:OH	+	++
	4	*E*7-12:Ac	-	-
	5	*Z*9-14:Ac	++++	+++
	6	*Z*11-16:Ald	++	+++
	7	*Z*11-14:Ac	++	+++
	8	***Z*** 9*E*11-14:Ac *	++++	++
	9	*Z*11-16:Ac	+	+
B	10	11-12:Ac	++	++++
	11	12:Ac	+++	+++
	12	*Z*9-12:Ac	++	++
	13	*Z*9*E*12-14:OH	+	+
	14	*E*11-14:Ac	+	++++
	15	*Z*9*E*12-14:Ac	+++	++
	16	***Z*** 9*Z*12-14:Ac *	+++	++
	17	14:Ac	++	+++

^1^ Strength of EAD responses: - no; + weak; ++ moderate; +++ strong; ++++ very strong. * Two female sex pheromone components of *Spodoptera litura*.

## Data Availability

The data presented in this study are available on request from the corresponding author due to the policy of the Rural Development Administration (Republic of Korea).
